# TRIAC Therapy Relieves Hyperthyroid Symptoms, Lowering T4, T3, and Metabolic Rate in Resistance to Thyroid Hormone β

**DOI:** 10.1210/clinem/dgaf583

**Published:** 2026-03-17

**Authors:** Carla Moran, Julie Martin-Grace, Greta Lyons, Laura Watson, Kevin Taylor, Susan Oddy, David Halsall, Krishna Chatterjee

**Affiliations:** 1 Endocrinology Section, Beacon Hospital, Dublin D18 AK68, Ireland; 2 Endocrinology Department, St. Vincent’s University Hospital, Dublin D04 T6F4, Ireland; 3 School of Medicine, University College, Dublin D04 V1W8, Ireland; 4 Wellcome-MRC Institute of Metabolic Science, University of Cambridge, Cambridge CB2 0QQ, UK; 5 Metabolic Research Area, NIHR Cambridge Clinical Research Facility, Cambridge CB2 0QQ, UK; 6 Department of Blood Sciences, Addenbrooke’s Hospital, Cambridge CB2 0QQ, UK

**Keywords:** thyroid hormone analogue, triiodothyroacetic acid (TRIAC), resistance to thyroid hormone beta

## Abstract

**Context:**

The treatment of resistance to thyroid hormone β (RTHβ) is challenging because features of hyperthyroidism in some tissues coexist with a hormone-resistant, hypothyroid state in other organs.

**Objective:**

To determine whether triiodothyroacetic acid (TRIAC) therapy alleviates hyperthyroid symptoms and changes circulating thyroid hormones, resting energy expenditure and metabolic parameters in RTHβ.

**Design:**

Retrospective cohort study.

**Setting:**

Referral center.

**Participants:**

Eight adult patients with RTHβ (mean age 41 years; 4 males, 4 females).

**Main Outcome Measures:**

Hyperthyroid Symptom Scale (HSS) score, serum free T4 and total T3, resting energy expenditure, sleeping heart rate, fasting lipids, and glucose and systemic insulin resistance.

**Results:**

From abnormally elevated levels prior to treatment, TRIAC therapy lowered HSS scores (baseline 17.5 vs posttreatment 6; *P* = .0014), showed a reduced resting energy expenditure trend (baseline Z-score +1.3 vs posttreatment +0.89; *P* = .38) and normalized circulating free T4 (baseline free T4 30 pmol/L vs posttreatment 17 pmol/L; *P* = .007) and total T3 (baseline 2.5 nmol/L vs posttreatment 1.4 nmol/L; *P* = .001) concentrations in 7 out of 8 patients, without any rise in their serum TSH (baseline 2.1 mU/L vs posttreatment 1.7 mU/L; *P* = .65), total cholesterol (baseline 4.9 mmol/L vs posttreatment 4.8 mmol/L; *P* = .81), and triglyceride (baseline 1.3 mmol/L vs post-treatment 1.3 mmol/L; *P* = .44). Their mean sleeping heart rate (baseline 60 bpm vs posttreatment 58 bpm; *P* = .56) and plasma N-terminal pro-brain natriuretic peptide (baseline 55 ng/L vs posttreatment 54 ng/L) were unchanged. TRIAC treatment was well tolerated, with no side effects.

**Conclusion:**

TRIAC therapy in RTHβ relieves hyperthyroid symptoms and lowers resting energy expenditure and circulating thyroid hormones without worsening hepatic hormone resistance or exacerbating cardiac thyromimetic activity. Future clinical trials to determine whether TRIAC treatment alters adverse cardiovascular outcomes in this disorder are warranted.

Resistance to thyroid hormone β (RTHβ), typically due to heterozygous mutations in thyroid hormone receptor β (TRβ) (1), with the mutant receptor inhibiting function of its wildtype counter-part (2, 3) in TRβ-expressing tissues (eg, hypothalamic thyrotropin releasing hormone neurons, pituitary thyrotrophs), results in elevated circulating thyroid hormones (TH) and non-suppressed TSH levels (4).

Although many individuals with RTHβ are asymptomatic, patients can exhibit signs and symptoms of thyrotoxicosis. In infancy and childhood, symptoms can include tachycardia, hyperkinetic behavior, and failure to gain weight (5); features in adults include heat intolerance and sweating, anxiety and insomnia, peripheral tremor, palpitations, and difficulty maintaining body weight (6). Similar to conventional hyperthyroidism, heat intolerance, tachycardia, and tremor partly reflect adrenergic overactivity secondary to sympathovagal imbalance associated with TH excess (7). However, the cardiac hyperthyroidism (8) and raised metabolic rate (9) seen in RTHβ are also consequences of elevated circulating THs acting on tissues (eg, myocardium, skeletal muscle) that express normal, intact TRα. Indeed, the occurrence of marked thyrotoxic signs and symptoms in some RTHβ cases has previously led to some patients being classified as having predominant central (or pituitary) resistance to TH (6). Conversely, refractoriness to TH action in the liver, which expresses mainly TRβ, may in part mediate the dyslipidemia [raised low-density lipoprotein cholesterol (LDL), triglycerides] and hepatic steatosis (9–11) associated with RTHβ. The disorder is also associated with increased systemic insulin resistance (9, 10) and other features of metabolic syndrome (10).

Recognized cardiac manifestations of RTHβ include tachycardia and echocardiographic indices of cardiac contractility in the hyperthyroid range (8, 12). Recently, 3 studies of independent, large RTHβ cohorts have documented significantly increased cardiovascular risk and morbidity (atrial fibrillation, heart failure, and major adverse cardiovascular events) (13–15), with a reduction in life expectancy of RTHβ patients by a decade compared to the general population (13). Two of these studies showed that adverse cardiac events were more prevalent in patients with higher free T4 (FT4) levels (13, 14), raising the possibility that TH-lowering therapy may prevent such outcomes.

However, conventional antithyroid drug therapy or thyroid ablation (surgery, radioiodine) induces an exaggerated rise in circulating TSH, driving continued excess TH synthesis and goitre formation (16); moreover, following thyroid ablation, thyroxine replacement in supraphysiological dosage is required to prevent thyrotroph hyperplasia and pituitary enlargement (17). For these reasons, recent European Thyroid Association guidelines on the management of RTHβ recommend avoiding antithyroid drug treatment or thyroid ablation in this disorder (18).

3′3′5-triiodothyroacetic acid (TRIAC), a TH analogue with greater affinity for TRβ than for TRα and which preferentially activates some TRβ mutants (19), reduces TSH secretion and thereby lowers circulating TH concentrations (20) but is devoid of significant thyromimetic activity in peripheral tissues (21). Used either as monotherapy or in combination with β-blockade or antithyroid drugs (16, 22, 23), TRIAC is not currently licensed for treatment of RTHβ but can be prescribed for selected, eligible patients under the auspices of its manufacturer’s Managed Access Policy (https://www.egetis.com/pipeline/managed-access-policy/).

In a retrospective analysis, we report our real-world experience of treating adult RTHβ patients experiencing hyperthyroid symptoms with TRIAC. We have delineated clinical, biochemical, and metabolic changes following TRIAC therapy and monitored for any adverse effects, including possible augmentation of the thyrotoxic state in TRα-expressing tissues and worsening of hormone resistance and the relative hypothyroid state of TRβ-expressing target organs.

## Methods

### Patients and Healthy Control Participants

Eight adult patients with RTHβ, harboring diverse TRβ mutations, who had been referred to our center were included in this retrospective cohort study if they were being treated with TRIAC monotherapy (Emcitate^®^; Tiratricol) to control hyperthyroid symptoms via a compassionate use, managed access program established by its manufacturer (Egetis Therapeutics, Stockholm, Sweden), instituted in our center in 2014. All investigations in patients and healthy control participants (160 adults, age 17-65 years) were undertaken as part of protocols approved by Local Research Ethics Committees (RTH: Cambridgeshire Local Research Ethics Committee, LREC 98/154; Healthy control subjects: Cambridge Central Research Ethics Committee, REC 06/Q0108/84), with prior informed, written consent of patients and healthy subjects.

Prior to and periodically during TRIAC treatment, patients were assessed in the NIHR Cambridge Clinical Research Facility. Each visit consisted of clinical assessment including completion of the Hyperthyroid Symptom Scale (HSS) questionnaire and measurement of body composition, resting energy expenditure (REE), sleeping heart rate, and biochemical parameters following a supervised overnight fast. Except in patient P5, β-blocker therapy was discontinued for at least 72 hours prior to the assessment of patients.

### HSS Questionnaire

Patients completed the HSS, a validated questionnaire that rates thyrotoxic symptoms from 0 to 4 using a 10-item inventory that includes nervousness, sweating, heat intolerance, hyperactivity, tremor, weakness, tachycardia, diarrhoea, appetite, and impairment of daily function, with higher scores representing greater symptom burden (highest score 40). Euthyroid individuals have reported HSS scores of ≤7, with scores of ≥20 being reported in subjects with conventional thyrotoxicosis (24).

### Resting Energy Expenditure and Heart Rate

REE, in patients or healthy control subjects, was measured by indirect calorimetry, calculated from corrected gas exchange volumes (Gem Nutrition, Daresbury, UK) using the derivations of Elia and Livesey (25). Body composition and bone mineral density were measured by dual energy x-ray absorptiometry (Lunar iDXA, encore V.18). Precision of the instruments was 0.4% coefficient of variation for lean mass and 3.8% coefficient of variation for REE (26, 27). REE was adjusted for lean body mass and expressed as a SD or Z-score relative to a healthy, control population REE dataset as described previously (28, 29). A resting electrocardiogram was performed at each visit to assess heart rate and rhythm. Actiheart (CamnTech, Fenstanton, Cambridgeshire, UK; software version 4.0.116 and 5.1.29), a device attached to the precordium that has been validated in human subjects (30), recorded sleeping heart rate.

### Biochemical Measurements

Serum TSH (Siemens Catalog # 10995703, RRID: AB_2895183), free T4 (Siemens Catalog # 10995589, RRID: AB_2895179), free T3 (Siemens Catalog # 10995584, RRID: AB_3675939), N-terminal pro-brain natriuretic peptide (NT-proBNP; Siemens Catalog # 11200588, RRID: AB_3714838), SHBG (Siemens Catalog # L2SH12, RRID: AB_2750986), and lipid profiles were measured using assays on Atellica IM or CH analyzers (Siemens Healthineers, Erlangen, Germany). Total T4 and T3 were quantified using an in-house, liquid chromatography tandem mass spectrometry (LC-MS/MS) method that selectively measures these iodothyronines in the presence of TRIAC. Nonesterified fatty acids were measured by Roche Free Fatty Acids (Roche Diagnostics, Mannheim, Germany); and insulin by Diasorin XL Liason (DiaSorin Catalog # 310360, RRID:AB_3099584) methods, with calculation of Homeostatic Model Assessment of Insulin Resistance (HOMA-IR) from fasting plasma glucose and insulin concentrations using the formula: HOMA-IR = fasting plasma glucose (mmol/L) × fasting plasma insulin (mU/L)/22.5 as described previously (10).

### Statistical Analysis

For the parameters specified earlier (biochemical measurements, REE, sleeping heart rate), we compared baseline, pre-treatment values with measurements at the timepoint when circulating FT4 was at its lowest during TRIAC therapy (nadir FT4). However, due to the relatively short interval between baseline and nadir FT4 timepoints, comparisons of bone mineral density (BMD) and body mass index were between pre-treatment values and measurements at their most recent assessment visit on TRIAC therapy.

Statistical analysis was performed using GraphPrism 10.1.0 for Windows (GraphPad by Dotmatics, San Diego, CA, USA). Data were expressed as counts (%) for categorical variables and as means (SD) and medians (IQR) for continuous variables for normally and nonnormally distributed data, respectively. Comparisons of data pre- and post-TRIAC treatment were made using the Mann–Whitney *U*-test or paired *t*-tests for nonparametric and parametric data, respectively. A *P*-value of <.05 was considered statistically significant.

## Results

### Patient Characteristics

Eight patients [4 males, 4 females; median age 32 years (range 18-55 years), whose baseline characteristics and symptoms are described in Table 1 and Table S1 (31), were studied. At the time of analysis, patients had been on TRIAC treatment at an average dose of 1.8 mg (range 1.3-3 mg), divided equally between twice- or thrice-daily regimens (Table 1), for a median of 40 months (range 13-143 months), with all but 1 patient remaining on therapy.

**Figure 1:**
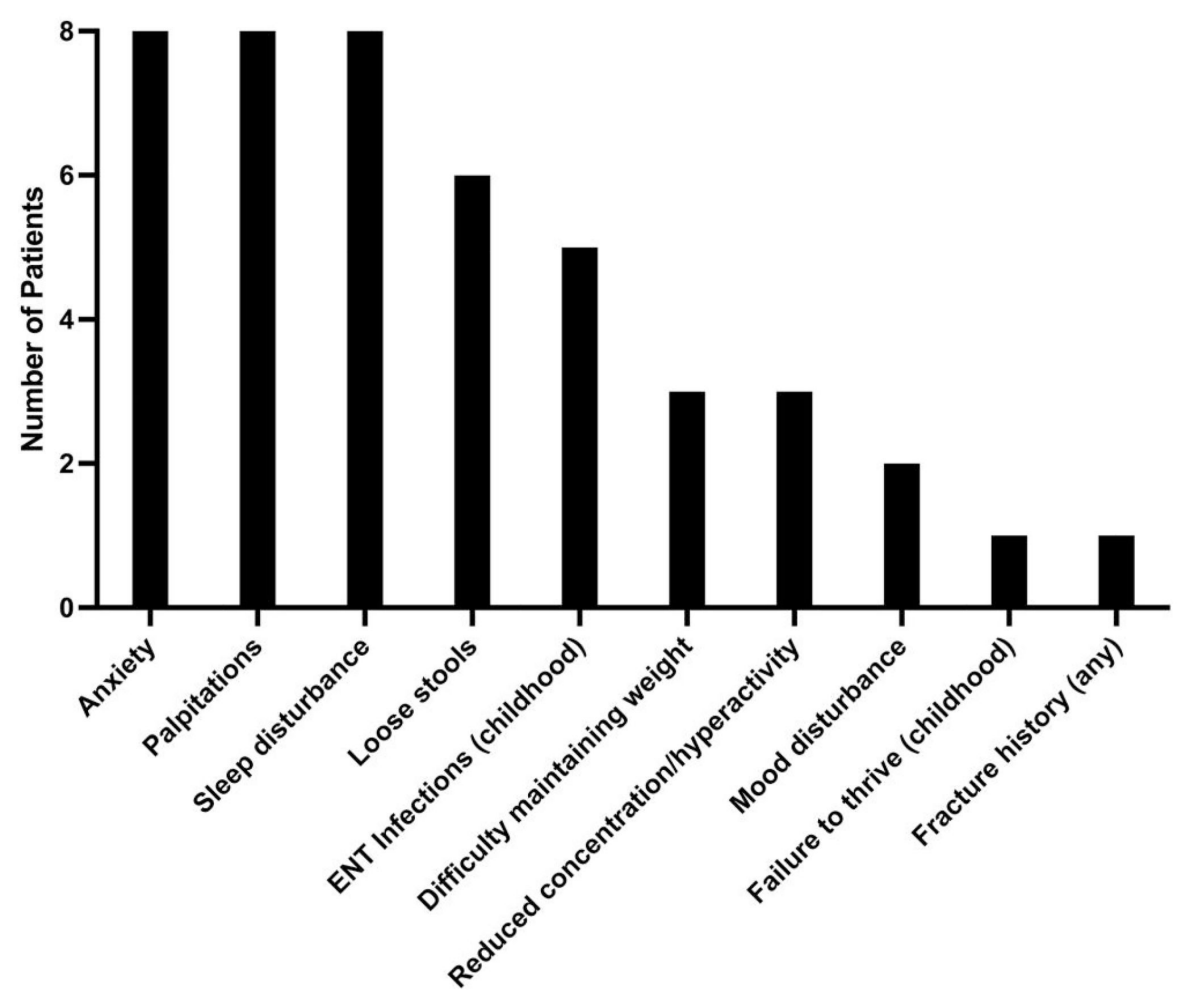
Symptoms reported by participants at baseline.

Prior to TRIAC treatment, patients reported a constellation of symptoms including anxiety, palpitations, sleep disturbance, loose stools, and difficulty maintaining weight. Several patients reported recognized features of RTHβ (ear, nose and throat infections, reduced concentration or hyperactivity, failure to thrive) in childhood (Fig. 1).

### Changes in HSS Score, Circulating THs, REE, and Heart Rate

**Figure 2:**
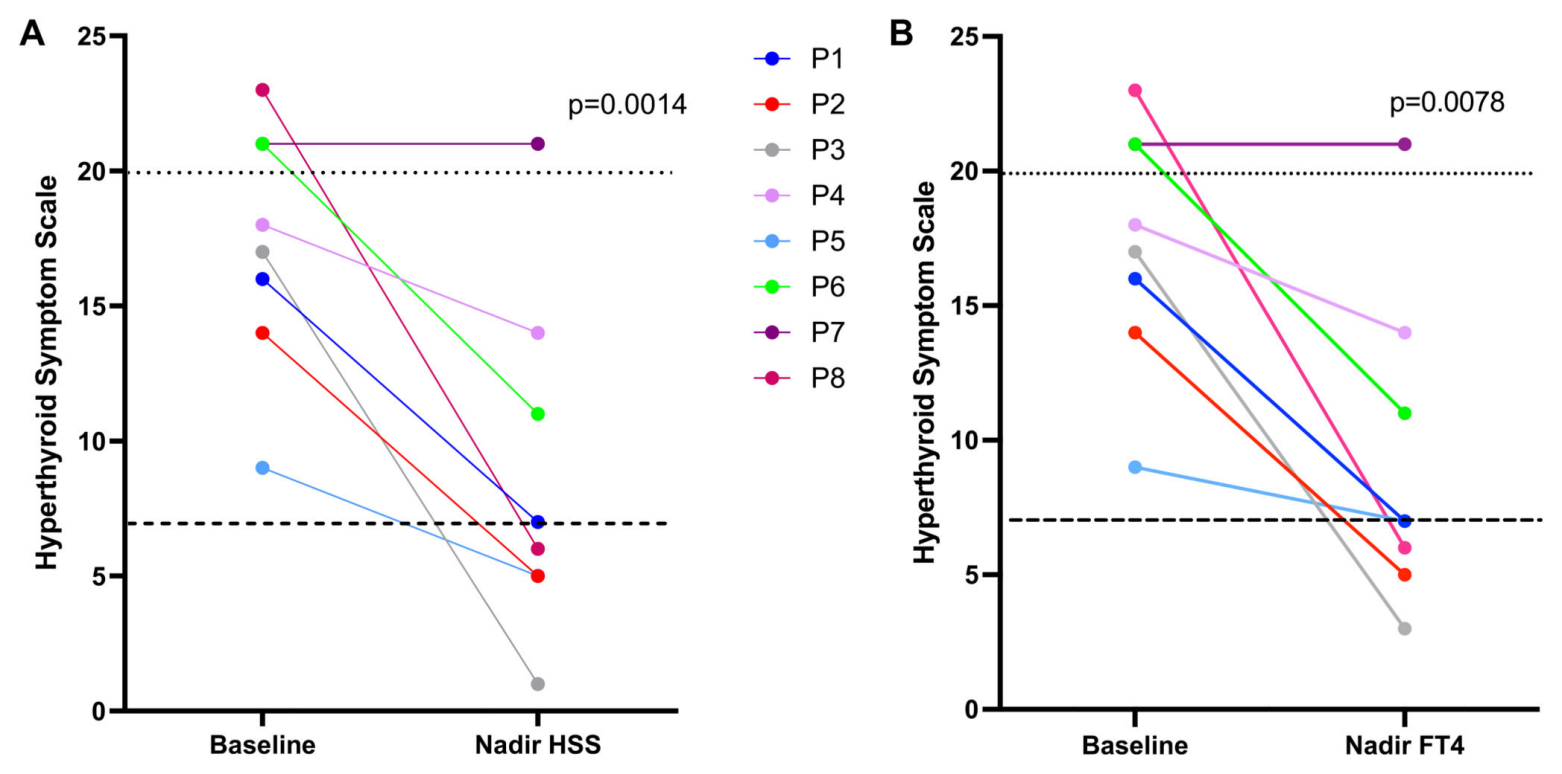
Comparison of HSS in RTHβ patients at baseline with their lowest HSS score achieved during TRIAC treatment (A) or their HSS score at the timepoint when their FT4 was lowest (nadir FT4) (B). The lower dashed line denotes HSS scores reported in euthyroid individuals (≤7) and the upper, dotted line denotes scores (≥20) reported in subjects with conventional thyrotoxicosis. Solid lines denote individual patients (P1-P8). Abbreviations: HSS, hyperthyroid symptom scale; RTHβ, resistance to thyroid hormone β; TRIAC, 3′3′5-triiodothyroacetic acid.

RTHβ patients showed elevated HSS scores (median 17.5, range 15-21) at baseline, with a fall in HSS scores following TRIAC treatment (Fig. 2), achieving a nadir score (median 6, range 5-11) that was significantly lower (*P* = .0014) than pretreatment values (Table 2). In 5 patients, taking TRIAC at a median dose of 1.4 mg/day (range 1.1-2.8 mg/day), nadir HSS scores fell into the euthyroid range (≤7) (Fig. 2A), with normalization occurring within a median of 26 months (range 5-80 months) of treatment.

**Figure 3:**
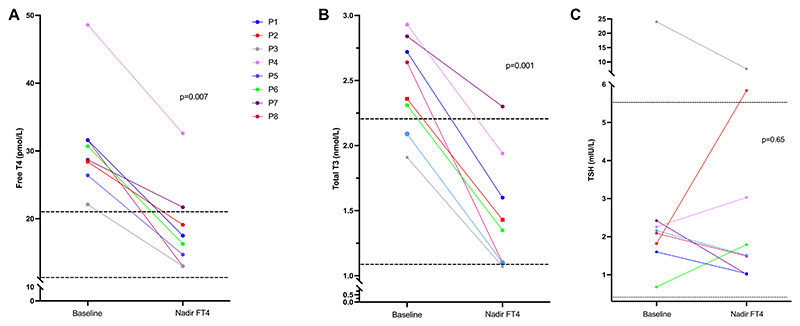
Changes in thyroid function tests in RTHβ patients from baseline compared with the posttreatment timepoint when FT4 was lowest (nadir FT4), showing values for FT4 (A), total T3 (B), and TSH (C). Dotted lines denote the upper and lower limits of reference intervals for each measurement (FT4 10.5-21 pmol/L, total T3 1.1-2.2 nmol/L, TSH 0.35-5.5 mIU/L). Solid lines denote individual patients (P1-8). Abbreviations: FT4, free T4; RTHβ, resistance to thyroid hormone β.

Prior to TRIAC treatment, raised serum free THs (median FT4 30, range 27-31 pmol/L; median FT3 9.9 pmol/L, range 8-10 pmol/L) and total T3 (median TT3 2.5 nmol/L, range 2.1-2.8 nmol/L) concentrations, with nonsuppressed TSH levels (median TSH 2.1 mIU/L, range 1.8-2.4 mIU/L) in patients (Tables 1 and 2), were consistent with the diagnosis of RTHβ. Analyzing biochemical outcomes at the timepoint (nadir FT4) when circulating FT4 reached its lowest concentration after treatment, significant reductions in FT4 (baseline 30 pmol/L vs posttreatment 17 pmol/L, *P* = .007, Table 2, Fig. 3A) and total T3 concentrations (baseline 2.5 nmol/L vs posttreatment 1.4 nmol/L, *P* = .001, Table 2, Fig. 3B) were recorded, with no corresponding rise in TSH levels (baseline 2.1 mIU/L vs post-treatment 1.7 mIU/L, *P* = .65, Table 2, Fig. 3C), after TRIAC therapy of patients. TRIAC treatment, in median dosage of 1.4 mg/day (range 1.4-2.8 mg), for an average duration of 8 months (range 4-33 months), normalized circulating FT4 concentrations in 88% (7 out of 8) of cases. Patient P4 elected to take other concurrent medication [propranolol, mirtazapine, melatonin; Table S1 (31)], to control his symptoms rather than increase the dosage of his TRIAC therapy to lower his serum FT4, which had not fully normalized (Fig. 3A).

**Figure 4:**
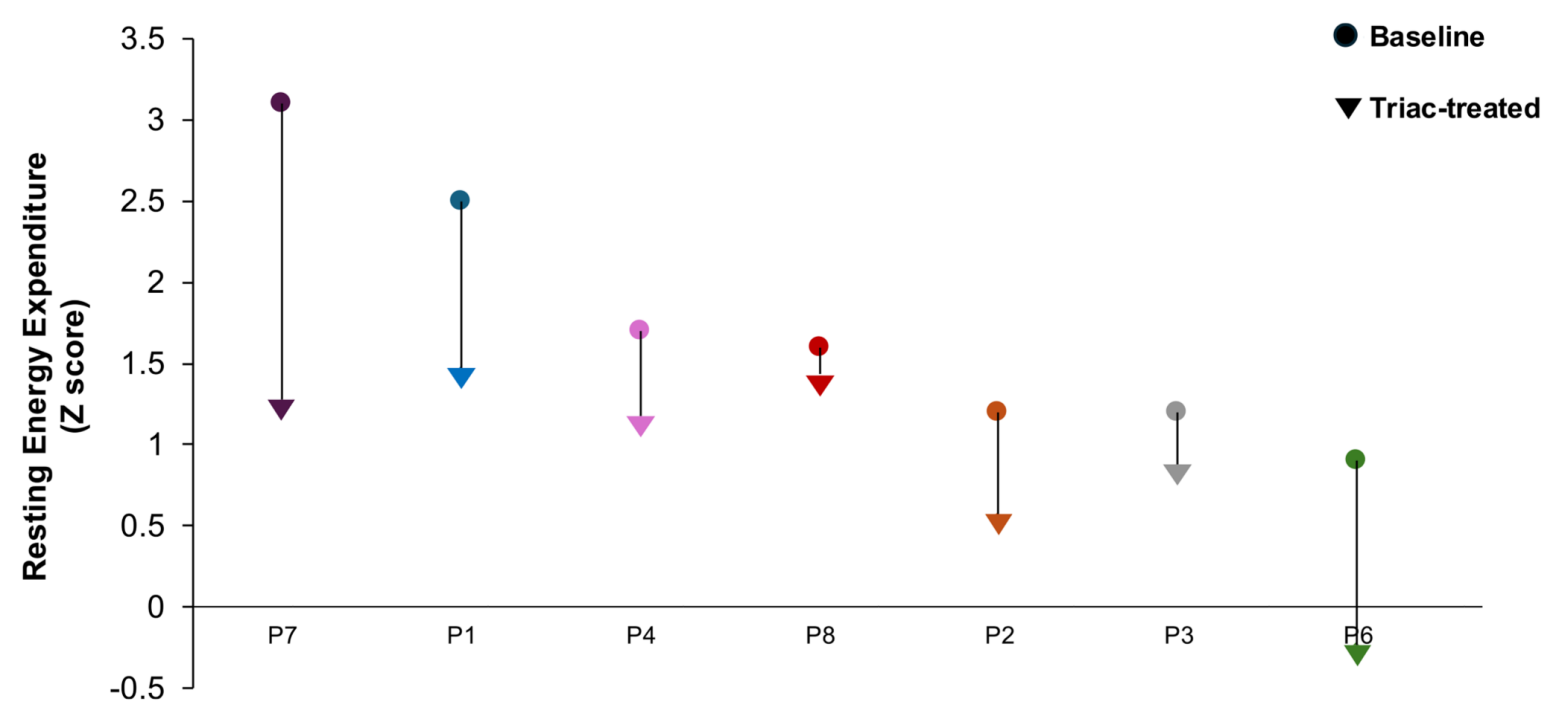
Changes in resting energy expenditure in each RTHβ patient (P1-P8), comparing measurements at baseline with the timepoint when their FT4 was lowest (nadir FT4), with values expressed as a Z score compared to healthy adult subjects. *REE comparator taken at nadir HSS timepoint for P1. REE was not measured in P5, who was unable to discontinue β-blocker therapy. Abbreviations: FT4, free T4; REE, resting energy expenditure; RTHβ, resistance to thyroid hormone β.

At baseline, REE was higher in RTHβ patients than healthy controls (mean baseline Z-score +1.3), with values falling after TRIAC therapy (posttreatment Z score +0.89, *P* = .38, Table 2, Fig. 4).

There was no significant change in either sleeping heart rate (baseline, 60 bpm vs 58 bpm, posttreatment, *P* = .56) or plasma NT-proBNP concentrations (baseline 55 ng/L vs 54 ng/L posttreatment, *P* = .99) after TRIAC therapy [Table 2, Fig. S1 (31)]. Throughout the study period, all patients remained in normal sinus rhythm.

### Body Weight, Lipid Profiles, Systemic Insulin Sensitivity, and BMD

A small, nonsignificant increase in body mass index of patients (median baseline, 23.9 kg/m^2^ vs median, posttreatment 24.4 kg/m^2^, *P* = .85) was recorded following TRIAC treatment (Table 2). Excluding 1 patient (P3) who commenced atorvastatin during the study period from analysis, we observed no significant changes in serum total cholesterol, LDL, high-density lipoprotein, or triglyceride concentrations of patients when values of these parameters at baseline vs at the posttreatment, nadir FT4, timepoint were compared [Table 2, Fig. S2 (31)]. Fasting plasma glucose and HOMA-IR (an index of whole-body insulin sensitivity) were also unchanged following TRIAC therapy [Table 2, Fig. S2 (31)]. SHBG concentrations rose slightly (median baseline 30 nmol/L vs median, posttreatment 41 nmol/L), but this change was not significant (*P* = .13) [Table 2, Fig. S2 (31)].

There was no significant change in BMD at either the lumbar spine (baseline median Z-score, −0.74 vs posttreatment median Z-score −0.2, *P* = .77) or hip (baseline median Z-score −0.74 vs posttreatment median Z-score −0.64, *P* = .8) seen after varying periods (13-143 months) of TRIAC therapy in patients [Table 2, Fig. S3 (31)]. One patient (P3), in whom low bone density at baseline (age 55 years, lumbar spine T-score −2.2) deteriorated further at menopause (age 57 years, T score −3.6), was treated with bisphosphonate for 5 years, stabilizing her BMD (age 63 years, T score −2.9).

## Discussion

Consistent with their constellation of thyrotoxic symptoms, raised baseline HSS scores in our 8 RTHβ patients mirrored values seen in untreated Graves’ disease (24), with their symptoms significantly impairing daily function in 6 cases. Following TRIAC therapy, reductions in HSS scores signifying improved symptom burden were recorded in all patients, with scores normalizing to within the euthyroid range in 5 individuals. We conclude that TRIAC therapy was effective in relieving thyrotoxic symptoms of RTHβ patients, fulfilling the primary aim of such treatment in our patients.

Although we did not titrate TRIAC therapy to achieve a specific biochemical target, nadir FT4 concentrations fell to within the reference interval in all but 1 patient, with the lowest HSS scores recorded in each patient corresponding to timepoints when circulating FT4 concentrations were either at their lowest (n = 6) or normal (n = 1). We surmise that interindividual differences in hyperthyroid symptoms, concurrent β blockade, and responsiveness to TRIAC therapy could account for varying time intervals taken to normalize HSS scores or FT4 concentrations. As TRIAC cross-reacts in T3 immunoassays (23), such measurements cannot be used to monitor the biochemical efficacy of TRIAC therapy (18). Using an LC-MS/MS method that is not susceptible to such cross-reactivity (32), we have shown that serum total T3 concentrations fell significantly, normalizing in almost all patients after TRIAC therapy. These observations provide important proof-of-principle that measurements of circulating free T4 and total T3, using immunoassay or LC-MS/MS methods, respectively, can be used to monitor the efficacy of TRIAC therapy in RTHβ patients. Two previous reports have suggested that patients harboring TRβ mutations (V264D, R243Q) that localize to 1 mutation cluster are biochemically or clinically unresponsive to TRIAC (20, 33, 34). However, our experience of excellent biochemical and clinical responses to TRIAC therapy in patient P1, who is heterozygous for R243Q mutant TRβ, or in another homozygous R243Q case (16) suggests that refractoriness to TRIAC therapy may be mediated by factors unrelated to TRβ mutant genotype.

As we have documented previously in this disorder (9), the REE of 7 untreated RTHβ patients was higher than healthy controls, with TRIAC therapy reducing this in all cases. Our observations differ from increased REE following TRIAC treatment reported in 2 RTH patients (35) but accord with normalization of raised basal metabolic rate in another TRIAC-treated RTH case (36). Mean sleeping heart rate and plasma NT-proBNP (which can be elevated in some untreated RTHβ patients) (15) were unchanged in our TRIAC-treated RTHβ patients. These findings fit with unchanged cardiac parameters seen in patients with euthyroid goitre treated with TRIAC in TSH-suppressive dosage (37). Overall, the fall in REE together with unchanged heart rate and circulating NT-proBNP levels in our TRIAC-treated RTHβ patients suggests that this TH analogue lacks significant thyromimetic activity in TRα-expressing skeletal muscle and heart.

Even when TRIAC therapy had maximally reduced circulating TH concentrations (nadir FT4 timepoint) in RTHβ patients, their TSH levels did not rise significantly, consistent with the notion that this TH analogue has central thyromimetic activity. Furthermore, following TRIAC treatment, lower TH concentrations were not associated with elevation in circulating cholesterol and triglycerides, and levels of serum SHBG, a hepatic marker of TH action, rose slightly. Our observations are congruent with a previous study showing that, when administered to athyreotic patients, TRIAC lowered total and LDL cholesterol and raised SHBG, suggesting the analogue may have some hepatic thyromimetic activity (38). Overall, these findings indicate that TRIAC-induced reduction in circulating THs in RTHβ patients has not worsened hormone resistance and the relative hypothyroid status of TRβ-expressing tissues (eg, liver, pituitary).

In all patients, TRIAC therapy was well tolerated in all patients without any reported adverse effects. In 1 case (P1), TRIAC therapy resolved hyperthyroid symptoms so effectively that β blockade was discontinued, with subsequent resolution of β-blocker-associated symptoms (fatigue, somnolence, and mood disturbance), further improving his well-being. Conversely, 2 patients (P6, P7) chose to continue both β-blocker and TRIAC treatment, reporting that β blockade is particularly effective at controlling palpitations and tachycardia, with TRIAC therapy best alleviating other hyperthyroid symptoms. In conventional hyperthyroidism, β-adrenergic blockade is unable to reverse its adverse effect on cardiac isovolumic relaxation time (39). If the adverse cardiac outcomes seen in RTHβ are mediated by similar mechanisms (13, 14), this may provide a further rationale for combining β blockade with TRIAC therapy. Consistent with the known spontaneous remission of thyrotoxic symptoms in RTHβ (40), 1 patient (P2) elected to discontinue TRIAC therapy after 4 years of treatment and remains asymptomatic despite circulating THs rebounding to elevated, pretreatment levels.

We acknowledge that findings in this study, representing responses to TRIAC therapy in a very small group of RTHβ patients experiencing significant thyrotoxic symptoms, cannot necessarily be extrapolated to the wider, general RTHβ population. Indeed, with recent studies suggesting that adverse cardiac outcomes are linked to chronic exposure to high circulating TH in this disorder (13–15), larger studies including randomized controlled trials, examining whether normalizing circulating THs with TRIAC therapy alters cardiovascular outcomes in RTHβ, are warranted.

## Figures and Tables

**Table 1:** Characteristics of study cohort, dosage, and duration of TRIAC therapy

	Number	Range
Number	8	
Male, n (%)	4 (50)	
Remain on TRIAC treatment (last follow-up), n (%)	7 (88)	
Age (years; median, IQR)	36 (24–42)	20-68
Age at enrollment (years; median, IQR)	32 (21-38)	18-55
Duration of treatment (months; median, IQR)	40 (25-63)	13-143
Twice-daily regimen, n (%)	4 (50)	
Thrice-daily regimen, n (%)	4 (50)	
Total daily dose (mg; median, IQR)	1.8 (1.4-2.8)	1.05-3.5
Total daily dose (mg; median, IQR) (average over treatment period)	1.4 (1.2-2.4)	1-3.3
β-blocker coprescription, n (%)	6 (75)	
Baseline TSH (mIU/L; median, IQR) RR 0.35-5.5 mIU/L	2.1 (1.8-2.4)	0.68-24
Baseline FT4 (pmol/L; median, IQR) RR 10.5-21 pmol/L	30 (27-31)	22-49
Baseline FT3 (pmol/L; median, IQR) RR 3.5-6.5 pmol/L	9.9 (8-10)	6.2-16
Number of patients achieving FT4 within reference interval, n (%)	7 (88)	
Time taken to achieve FT4 within reference interval (months; median, IQR)	5 (4-33)	2-38
Dose required to achieve FT4 within reference interval (mg/day; median, IQR)	1.4 (1.4-2.8)	0.7-3.5
Number of patients who reached HSS score ≤7 (euthyroid range)	5/8 (63%)	
Time taken to achieve HSS score within euthyroid range (months; median, IQR)	26 (6.5-64)	5-80
Dose required to achieve HSS score within euthyroid range (mg/day; median, IQR)	1.4 (1.4-2.7)	1.1-2.8

Abbreviations: FT3, free T3; FT4, free T4; HSS, Hyperthyroid Symptom Scale; IQR, interquartile range; RR, reference range; TRIAC, 3′3′5-triiodothyroacetic acid.

**Table 2:** Changes in parameters before and after TRIAC therapy

	Baseline	After TRIAC treatment	*P*-value
HSS score (IQR)	17.5 (15-21)	6 (5-11)^ a^	.**0014**
Resting energy expenditure (Z-score)	1.3 (± 1.3)	0.89 (0.84)	.38
Sleeping heart rate (bpm) (IQR)	60 (52-65)	58 (54-60)	QR
NT-proBNP (ng/L) (IQR)	55 (35-124)	54 (35-159)	.99
BMI (kg/m^2^)^b^	23.9 (± 5.2)	24.4 (± 4.8)	.85
BMD lumbar spine (Z-score)^b^ (IQR)	-0.3 (0.06, -0.05)	-0.2 (-1.1 to 0.3)	.77
BMD total hip (Z-score)^ b^	-0.74 (± 0.76)	-0.64 (± 0.84)	.8
TSH (mIU/L) (IQR)	2.1 (1.8-2.4)	1.7 (1.1-5.1)	.65
FT4 (pmol/L) (IQR)	30 (27-31)	17 (13-21)	.**007**
Total T3 (nmol/L) (IQR)	2.5 (2.1-2.8)	1.4 (1.1-1.9)	.**001**
Total cholesterol (mmol/L)	4.9 (± 1.5)	4.8 (± 1.1)	.81
LDL cholesterol (mmol/L)	2.7 (± 0.84)	2.9 (± 0.9)	.75
HDL cholesterol (mmol/L)	1.1 (± 0.32)	1.2 (± 0.21)	.38
Triglycerides (mmol/L) (IQR)	1.3 (1.2-1.9)	1.3 (1-1.4)	.44
Fasting glucose (mmol/L)	4.9 (± 0.77)	4.8 (± 0.51)	.65
Fasting insulin (pmol/L)	51 (± 31)	51 (± 26)	.99
HOMA-IR	1.7 (± 1.3)	1.6 (± 0.8)	.82
HbA1c (mmol/mol)	34 (± 3.1)	34 (± 3.3)	.82
SHBG (nmol/L)	30 (± 14)	41 (± 14)	.13
NEFA (umol/L)	365 (± 219)	366 (± 198)	.99

Unless specified otherwise, values shown are mean ± SD, with comparisons between values at baseline and timepoint when posttreatment FT4 was lowest (nadir FT4). Statistically significant changes in parameters are shown in bold.

a Comparison between baseline and lowest, self-reported, posttreatment HSS score.

b Comparison between baseline and most recent study visit.

Abbreviations: BMI, body mass index; BMD, bone mineral density; HbA1c, hemoglobin A1c; HDL, high-density lipoprotein; HOMA-IR, Homeostatic Model Assessment of Insulin Resistance; HSS, Hyperthyroid Symptom Scale; IQR, interquartile range; LDL, low-density lipoprotein; NEFA, nonesterified fatty acid; NT-proBNP, N-terminal pro-brain natriuretic peptide; TRIAC, 3′3′5-triiodothyroacetic acid.

## Data Availability

Some or all datasets generated during and/or analyzed during the current study are not publicly available but are available from the corresponding author on reasonable request.
